# The Two-Faced Potato Virus X: From Plant Pathogen to Smart Nanoparticle

**DOI:** 10.3389/fpls.2015.01009

**Published:** 2015-11-17

**Authors:** Chiara Lico, Eugenio Benvenuto, Selene Baschieri

**Affiliations:** Laboratory of Biotechnology, ENEA, Rome, Italy

**Keywords:** potato virus X, nanotechnology, plant virus, nanomedicine, nanoparticles

## Abstract

Potato virus X (PVX) is a single-stranded RNA plant virus, historically investigated in light of the detrimental effects on potato, the world’s fourth most important food commodity. The study of the interactions with cells, and more generally with the plant, both locally and systemically, significantly contributed to unveil the mechanisms underlying gene silencing, fundamental not only in plant virology but also in the study of gene expression regulation. Unraveling the molecular events of PVX infection paved the way for the development of different viral expression vectors and consequential applications in functional genomics and in the biosynthesis of heterologous proteins in plants. Apart from that, the ease of manipulation and the knowledge of the virus structure (particle dimensions, shape and physicochemical features) are inspiring novel applications, mainly focused on nanobiotechnology. This review will lead the reader in this area, spanning from fundamental to applied research, embracing fields from plant pathology to vaccine and drug-targeted delivery, imaging and material sciences. Due to the versatile moods, PVX holds promise to become an interesting nanomaterial, in view to create the widest possible arsenal of new “bio-inspired” devices to face evolving issues in biomedicine and beyond.

## Introduction to Potato Virus X Molecular Pathology

Potato virus X (PVX) belongs to the genus *Potexvirus* of the *Flexiviridae* family that groups viruses phylogenetically related by replication mechanisms, structural proteins and genome type and organization (Martelli et al., 2007). PVX is mechanically transmitted and its main hosts are herbaceous plants, especially *Solanaceae*. It has been included among the pathogens with important economic impact and its effects can be worsened by co-infection with other viruses, in particular Potato virus Y (PVY; a phenomenon also known as “synergism”; Syller, 2012).

**FIGURE 1:**
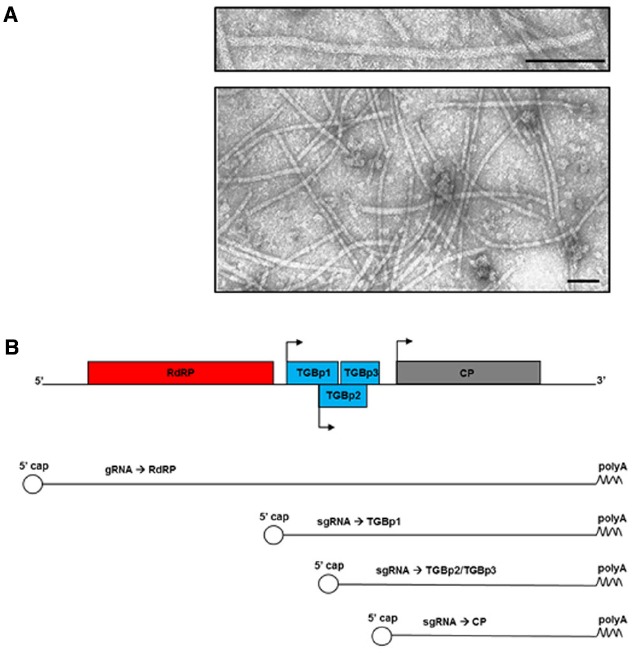
**(A)** Transmission electron micrographs of purified PVX nanoparticles (personal unpublished data). Bars: 100 nm. **(B)** Schematic diagram of PVX genome, genomic and subgenomic RNAs (gRNA and sgRNA, respectively), and the proteins they encode. RdRP: RNA-dependent RNA Polymerase; TGBp1/p2/p3: Triple Gene Block proteins; CP: Coat Protein. Arrows represent the subgenomic promoters. The 5′ cap and the polyA tail are indicated.

Potato virus X has a simple filamentous flexible structure of about 500 nm in length and 15 nm in diameter (Figure 1A). The virion has helical symmetry and a deeply grooved, highly hydrated surface (Parker et al., 2002). It is made of a single-stranded positive-sense RNA genome of approximately 6.4 kb (Huisman et al., 1988; Skryabin et al., 1988), wrapped in approximately 1300 units of a single coat protein (CP) type, with 8.9 CP units per helix turn (Kendall et al., 2008).

The genome is capped at the 5′-end and poly-adenylated at the 3′-terminus. It contains five open reading frames (ORFs) encoding five proteins: the RNA-dependent RNA Polymerase (RdRP), the movement proteins encoded by three overlapping ORFs that form the Triple Gene Block module (TGBp1, TGBp2, and TGBp3), and the CP (Figure 1B; Sonenberg et al., 1978; Morozov et al., 1987, 1991). The majority of these proteins are multifunctional and involved in several different steps of the infection process, together with numerous host factors. The RdRP, for example, contains a methyl transferase type 1 domain involved in viral RNAs capping (Rozanov et al., 1992), a helicase domain fundamental for viral replication (Davenport and Baulcombe, 1997), and a replicase domain (Longstaff et al., 1993); while TGBp1 has RNA-binding, ATPase (Leshchiner et al., 2006) and RNA-helicase (Kalinina et al., 2002) activities. Moreover, TGBp1 contributes to: (a) increase the size exclusion limit of the plasmodesmata (PD; Angell et al., 1996; Howard et al., 2004), (b) translationally activate the progeny virions (Karpova et al., 2006), and (c) counteract gene silencing (Voinnet et al., 2000), the plant innate mechanism of immune resistance.

When PVX enters plant cells, the particles unpack thanks to the phosphorylation events mediated by cellular enzymes on Ser/Thr residues located at the solvent-exposed N-terminus of the CP (Atabekov et al., 2001). The RdRP is directly synthetized from the viral RNA genome, exploiting the host translational machinery, to subsequently give rise to subgenomic RNAs and viral proteins (Batten et al., 2003). Later on, within infected cells, the replication complexes become part of structures called X-bodies that are formed next to the nucleus (Tilsner et al., 2012). X-bodies are isolated environments delimited by remodeled cellular membranes and organized in “compartments” that incorporate both viral and host components, coordinating the different phases of infection (replication, protein synthesis, assembly, and movement). The primary function of X-bodies is to protect and allow viral replication through the anchoring of RdRP in arrays to membranes, yet being sites where ribonucleoprotein (RNP) complexes (involved in cell-to-cell transfer of infection) and complete virus particles are assembled. TGBp1 has a central role in the formation of X-bodies as it participates to the remodeling of host actin filaments, endoplasmic reticulum (ER) and Golgi membranes, and to the recruitment of the viral TGBp2 and TGBp3. Within X-bodies, aggregates of TGBp1 and actin microfilaments wrap in spirals to form the typical “beaded sheets,” electron microscopy-visible structures in PVX infected cells (Linnik et al., 2013). The spirals are surrounded by naked viral RNAs and fine membrane hoops/granular vesicles that contain TGBp2, TGBp3, ribosomes, and viral replicase, probably representing replication sites. The TGBp2 and TGBp3 membrane-spanning proteins contribute individually and/or synergistically to the formation of these vesicles by remodeling the ER membranes. At the periphery of X-bodies, assembled viral particles accumulate.

In the initial/middle phases of the infection, TGBp2 and TGBp3 exploit the dynamic ER/actin network to direct the transfer of RNP complexes, made of viral RNA and CP, from the perinuclear to the cortical ER (Tilsner et al., 2012). It has been recently suggested that later on the TGBp2 and TGBp3 remodeled ER-membranes form caps of the PD orifice and that these caps, harboring the RdRP and viral RNA, become the viral replication sites. According to this “co-replication model,” as soon as they are synthetized, RNP complexes are inserted by the TGBp1 into the PD for cell-to-cell transfer (Tilsner et al., 2013).

As for viral spread throughout the plant, the CP is necessary both in cell-to-cell and in systemic movement, but recently published data indicate that virus particle assembly is dispensable for local while essential for long-distance transfer (Santa Cruz et al., 1998; Betti et al., 2012). The CP has a pivotal role in virus particle morphology and stability, and strongly influences the efficiency of infection spreading (Lico et al., 2006). Unfortunately, atomic-resolution data defining its folding are not available. A recent revision of the protein model indicates that it contains seven α-helices and six β-strands and confirms that the N-terminus is exposed on the viral surface (Baratova et al., 2004; Lukashina et al., 2009, 2012).

Potato virus X has been used as model to study plant-virus interactions and to elucidate the plant-defense mechanisms and viral countermeasures such as suppression of silencing (Ruiz et al., 1998; Ratcliff et al., 1999). The plant is not a passive host during viral infection and viral RNA sensing induces the activation of defense responses that involve a complex set of proteins. The post-transcriptional RNA gene-silencing (PTGS) is a homology-dependent gene inactivation strategy that is triggered by the unusual presence in the plant cell of double stranded (ds) RNAs (Tenllado and Díaz-Ruíz, 2001). In the case of a viral infection, the PTGS response is activated by highly structured regions in the viral genomes or in the viral replication intermediates. These abnormal RNA structures are digested by a plant cell dsRNA-specific RNase, named Dicer, into 21–24 nucleotides RNA species (small interfering RNAs, siRNAs; Hamilton and Baulcombe, 1999). The siRNAs are then exploited through a sequence complementarity mechanism to recruit the homologous RNA into an RNA-induced silencing complex (RISC), where it is digested (Voinnet, 2005).

Also viral proteins contribute to trigger plant innate defense. In particular, the CP has been shown to work as pathogen avirulence effector of the Rx protein-mediated resistance (Bendahmane et al., 1995). Folding of the Rx protein is modified by CP binding and consequently nucleotide-binding site leucine-rich domains are exposed thus determining the activation of the signaling cascade and cell death (Moffett et al., 2002; Rairdan and Moffett, 2006).

Like other plant viruses, PVX has evolved mechanisms to counteract plant innate defense through one of its multifunctional protein, the TGBp1. The protein inhibits the formation of the effector complexes involved in RNA silencing (Bayne et al., 2005) by inducing, through the proteasome pathway, the degradation of the Argonaute 1 nuclease, one of the RISC components (Chiu et al., 2010).

Also the PVX-*Potyviruses* synergistic interaction that results in the enhancement of pathogenicity of PVX in *Nicotiana benthamiana* plants is somehow related to virus-induced gene silencing through the involvement of the *Potyviruses* P1/Helper component proteinase (HC-Pro; González-Jara et al., 2005; Pacheco et al., 2012).

To bring over these fundamental studies aimed to clarify the complex mechanisms underlying the plant-virus interaction, a number of viral expression vectors have been developed (Lico et al., 2008; Hefferon, 2012; Gleba et al., 2014; Peyret and Lomonossoff, 2015). First generation viral expression vectors usually harbor the cDNA form of the complete viral genome, sometimes engineered to easily insert foreign sequences as additional ORFs, or in substitution of a viral one, in association to a strong promoter (Chapman et al., 1992). These vectors can be directly used to infect the plant hosts, when the viral genome is under the control of a plant specific promoter, or used as template to generate *in vitro* an infectious transcript. In second-generation vectors, viral components are separately inserted into binary vectors and used to transform independently different *Agrobacterium* cells. Bacterial cells are then mixed together and used to co-infiltrate plant leaves (Gleba et al., 2014). Most of these vectors have found application in different technological fields.

In functional genomics studies, they are used to vehicle an endogenous gene fragment, triggering the specific suppression of the corresponding sequence in the genome (Ruiz et al., 1998; Baulcombe, 1999). This reverse genetics technique known as virus-induced gene silencing, is a high-throughput approach to the analysis of plant gene functions.

In “molecular farming,” PVX-based vectors are currently being used to vehicle and induce the expression in plants of foreign genes encoding high added-value biomolecules (Komarova et al., 2010).

Last, but only in the order of appearance list, these vectors appear extremely appealing also for nanotechnologies (Lico et al., 2013), as described in the following paragraphs.

## PVX Nanoparticles for Subunit Vaccine Delivery

Subunit vaccines are formulations based on isolated pathogens components (proteins or peptides) that allow the activation of highly specific and protective immune responses. Nowadays, these vaccines are typically produced by recombinant DNA technologies. Compared to traditional inactivated or attenuated vaccines, subunit vaccines guarantee selectivity, specificity, low toxicity, stability and reduced risk of undesired side effects (Rappuoli, 2007). Main limitation of these vaccines is that isolated proteins or peptides being small (<10 nm) are *per se* unable to stimulate complete immune responses (innate, antibody, and cell-mediated). Indeed, it has been established that the efficiency of antigen uptake by antigen presenting cells (APCs) is strictly related to antigen sizes, and the larger surfaces of particulate antigens improve the interaction with APCs (Bachman and Jennings, 2010). To increase subunit vaccine immunogenicity, it is thus important to arrange isolated antigens in larger particles (20–200 nm). This is possible by entrapping them with adjuvants or by favoring self-assembly in supramolecular structures, such as in the case of virus-like particles generated by the self-assembly of viral capsid proteins (Rosenthal et al., 2014). A further possibility consists in delivering the antigen in association to nanoparticles, such as liposomes.

**FIGURE 2:**
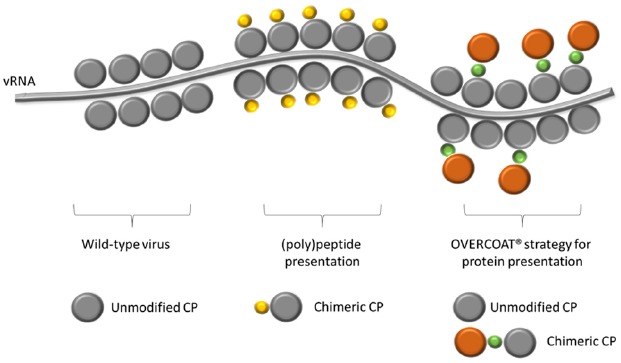
**Schematic representation of a PVX nanoparticle, showing the viral RNA (vRNA) and the shell composition of: a wild-type virion; a chimeric virus particle exposing on every single CP unit (in gray) a copy of the heterologous (poly)peptide (in yellow); a partially chimeric virus particle in which only some of the CP units are fused to an entire protein (in orange) through the OVERCOAT^®^ procedure.** In green the FMDV 2A peptide.

Many attempts have also been made to increase subunit vaccines immunogenicity and stability using genetically engineered plant virus nanoparticles as carriers for their delivery (Lico et al., 2007, 2013; Lebel et al., 2015; Streatfield et al., 2015). As for PVX, this provides for the fusion of the sequence encoding the immunogenic peptides or proteins in frame to the 5′-end of the gene encoding the CP in viral expression vectors. The N-terminus of the protein has indeed been demonstrated to be exposed on the external surface of the virion (Baratova et al., 1992). The modified viral expression vectors are then used to induce infection onset in plants and produce on large scale chimeric virus particles (CVPs) displaying on each CP subunit (approx. 1300 per virion) the (poly)peptide of interest (Figure 2). CVPs are then extracted from plant tissues and purified to be used in immunization trials.

Because the CP is involved in mediating the interaction with the plant environment, substantial modifications are not always tolerated in that they may interfere with both viral encapsidation and/or spread. These issues have been narrowly explored in the past years and some basic rules have been defined, whose compliance increases the chance of getting genetically stable infectious CVPs. Clearly, the shorter the exogenous (poly)peptide, the higher the probability that CP folding is preserved and compatible with efficient assembly. But length is not the unique parameter to be considered. Studies performed by fusing peptides (variable in length and amino acid composition) to a N-terminally truncated CP (a natural PVX mutant lacking the first 21 amino acids) have indicated that also isoelectric point, Ser/Thr and Trp content in the heterologous sequence may affect viral fitness (Lico et al., 2006). Recently, the insertion of a heterologous sequence between amino acids 23 and 24 at the N-terminus of the CP has also been reported (Vaculik et al., 2015). Nonetheless, up to now no CP domains other than the N-terminus have been identified that can harbor fusion peptides/proteins. Notably, the fusion to the C-terminus does not give rise to CVPs able to move throughout the plant (Cerovska et al., 2012). In fact, the C-terminus of the protein is crucial for the interaction with the viral RNA, hence for virion assembly (Nemykh et al., 2008).

On these bases, PVX particles displaying peptides of immunological relevance have been successfully produced in plants and their efficacy verified in animal models. PVX CVPs displaying the linear 2F5 epitope (2F5e) of the gp41 protein of Human Immunodeficiency Virus type 1 (HIV-1) envelope are able to induce the production of epitope-specific IgA and IgG, when administered intranasally or intraperitoneally in normal mice without the need to co-administer adjuvants. Notably, human dendritic cells pulsed with these CVPs are able to activate a 2F5e-specific human primary antibody response in severe combined immunodeficient mice engrafted with human lymphocytes. Finally, the antibodies induced by immunization with the CVPs are able to neutralize HIV-1 particles in viral neutralization assays (Marusic et al., 2001).

Potato virus X CVPs displaying a Major Histocompatibility Complex class I-restricted peptide of the nucleoprotein of influenza virus have also been engineered, taking into account mechanisms and limitations imposed by both plant virus biology and immune response. The synthetic DNA fragment encoding the influenza peptide was designed to be translated in an amino acid sequence optimized to: (i) obtain assembly of CVPs able to move systemically; (ii) minimize the risk of occurrence of unexpected mutations during viral replication; (iii) avoid detrimental effects of N-terminal modifications (such as acetylation) and improper C-terminal context on peptide processing by APCs. These rationally designed CVPs resulted to be genetically stable and triggered epitope-specific CD8^+^ T cells without the need of adjuvant co-delivery (Lico et al., 2009).

Overall, these data indicate that appropriately designed PVX CVPs are excellent epitope carriers endowed of adjuvant properties, probably not only because of the complex particulate structure but also because of the presence of the viral RNA that may trigger Toll-Like Receptor 7 on APCs (Jobsri et al., 2015). Altogether, these features account for the inherent ability of PVX to trigger a whole immune response, ideal condition to achieve complete protection from pathogens through vaccination.

Because one of the factors that may limit the construction of PVX CVPs is the length of the fused (poly)peptide, a “smart” procedure based on the OVERCOAT^®^ PVX vector has also been devised to avoid possible steric hindrance of chimeric CPs during particle assembly (Santa Cruz et al., 1996). To this aim, a 16 amino acid linker peptide (2A) derived from the Foot and Mouth Disease Virus (FMDV) has been inserted between the foreign sequence and the CP. During translation, this sequence may induce a ribosomal skip with a frequency that is dependent on the nature of the 18–30 amino acids in the region immediately upstream the 2A cleavage site (Minskaia and Ryan, 2013; Minskaia et al., 2013). The incomplete FMDV 2A-mediated “cleavage” results in the production of a heterogeneous population of CPs including original and engineered version, both contributing to the assembly of fully functional CVPs able to spread throughout the plant (Figure 2). By this strategy, PVX particles were constructed displaying on the surface different (poly)peptides of immunological interest. In the case of the rotavirus major inner capsid protein (VP6; O’Brien et al., 2000) and the tuberculosis ESAT-6 antigen (Zelada et al., 2006), data demonstrated only the structural stability of the CVPs and the correct display of the foreign sequences. Subsequent papers demonstrated also the ability of these PVX CVPs to induce antibody responses in animal models (rabbits and mice). This is the case of the CVPs tailored to display the D2 peptide from *Staphylococcus aureus* fibronectin-binding protein (Brennan et al., 1999), two peptides from the E2 glycoprotein of the classical swine fever virus (Marconi et al., 2006) or the R9 peptide from the hypervariable region I of hepatitis C virus (Uhde-Holzem et al., 2010).

Alternative approaches to the construction of PVX CVPs, even if at higher costs, imply standard chemical protein conjugation procedures. In this case, the success depends on the presence of free amine groups (Lys residues) and carboxylate groups (Asp and Glu residues). These amino acids are all present in PVX CP. Different bioconjugation protocols have been tested to functionalize PVX, revealing that the carboxylates groups are either not solvent-exposed or not accessible. On the other hand, N-Hydroxysuccinimide (NHS)-ester based and Cu(I)-catalyzed azide-alkyne 1,3-dipolar cycloaddition (“CLICK”) reactions on amine groups are both feasible (Steinmetz et al., 2010).

Potato virus X particles displaying a peptide derived from the extracellular domain of the Human epidermal growth factor receptor 2 (HER2) have been constructed by conjugation to Lys on the CP surface through a two-step reaction via a heterofunctional NHS-(poly)ethyleneglycole (PEG)_4_-maleimide linker (Shukla et al., 2014a). HER2 is an antigen usually expressed on breast cancer cells and known to induce the activation of B lymphocytes and antibody production. The chemically derivatized PVX particles were tested in mice and demonstrated to be effective in inducing the overcoming of HER2 immunological tolerance by Enzyme Linked ImmunoSorbent Assay and confocal microscopy using HER2-positive human cancer cells.

## PVX Nanoparticles for Imaging, Drug Delivery, and Diagnosis

Pioneering works in vaccine technology exploiting plant viruses such as PVX have worked as trailblazer in the field of nanotechnology. Indeed, the encouraging results prompted scientists in nanomedicine to broaden the exploration and exploitation of PVX particles as tools for targeted drug delivery or for diagnosis. In this perspective, the evaluation of the “biocompatibility” of viral particles, their natural tropism and biodistribution are fundamental to enter clinical trials. Indeed, the intrinsic toxicity cannot be taken for granted even if, for the animal cell, plant viruses are expected to behave as unreplicative and biologically safe nano-objects.

*In vitro* and *in vivo* experiments have been recently carried out to evaluate the toxicity and teratogenicity potential of unmodified PVX and Tomato Bushy Stunt Virus (TBSV), the type member of another viral genus and family, different in shape and dimensions (Blandino et al., 2015). Haemolysis assay was performed *in vitro* to determine detrimental effect of virus nanoparticles on human erythrocytes while toxicity and teratogenicity were evaluated *in vivo* through the early embryo assay (EEA). Chicken embryo is considered an excellent model to perform this kind of studies even if the reliability of the results depends on critical factors such as the choice of adequate controls, number of eggs per dose, and volume and route of delivery. Overall, the results of this study demonstrated that both plant viruses have neither cytotoxic effects on human erythrocytes nor toxic/teratogenic effects on chicken embryos. These data represent a good prerequisite for safety evaluation in view of forthcoming use of these nanoparticles as functionalized nanocarriers.

Biodistribution studies *in vivo* have been performed with PVX particles conjugated to various fluorescent dyes and to PEG with different chain lengths. PEG is frequently used to create a shield around biopharmaceuticals to increase solubility and stability and reduce immunogenicity even if, as a side effect, this masking reduces the interactions with cells, such as in the case of some plant virus nanoparticles (Raja et al., 2003; Schlick et al., 2005; Steinmetz and Manchester, 2009).

Preliminary studies *in vitro*, with human cervical cancer (HeLa) cells and murine fibroblasts (BalbCl7), highlight somewhat contradictory effects of PEGylation on PVX. In fact, this procedure effectively prevents interaction with cells of particles labeled with OregonGreen 488 (O488), while it promotes interaction if particles are labeled with Alexafluor 647 (A647; Steinmetz et al., 2010), and this is probably due to some intrinsic feature of the dye.

The analysis of the biodistribution and clearance of A647-labeled and PEGylated PVX in mice evidences that the viral nanoparticles remain mainly trapped by the mononuclear phagocyte system (MPS) that speeds up their removal from the circulation and from tissues. Nonetheless, the flexible filamentous PVX has enhanced tumor homing and tissue penetration, as compared to the icosahedral Cowpea Mosaic virus (Shukla et al., 2013, 2014b). Similarly, PVX CVPs constructed by the OVERCOAT^®^ procedure and carrying the mCherry fluorescent protein but not conjugated to PEG, were shown to accumulate in the liver and to co-localize with macrophages for final clearance from tissues via MPS, 7 days post-administration in mice (Shukla et al., 2014c). It has been recently shown that it is possible to significantly improve PVX half-life in the serum reducing tissue accumulation, renal clearance as well as uptake by immune cells through derivatization of the viral surface with a PEG chain tailored in length and conformation (brush vs. mushroom; Lee et al., 2015).

Beside these early studies aimed to gain insights on PVX nanoparticles-based delivery, advanced studies have already started to exploit the virus as scaffold for the conjugation of chemical moieties, constructing nano-bullets for molecular imaging and targeted drug delivery.

A647-labeled PVX nanoparticles, bioconjugated with a 12 amino acid peptide with affinity to the epidermal growth factor receptor (EGFR), are effective in detection and imaging of carcinoma cell lines that upregulate EGFR, and prefer partitioning to cancer cells rather than to macrophages (Chariou et al., 2015). Furthermore, PVX particles conjugated to the Herceptin (Trastuzumab) monoclonal antibody specific for the peptide HER2 have been demonstrated to be able to induce the apoptosis of HER2 positive cell lines (Esfandiari et al., 2015).

## Other PVX Nanoparticles Applications

Genetically or chemically modified PVX nanoparticles can be usefully applied also in fields other than vaccine and drug delivery or *in vivo* imaging.

A diagnostic kit for the early detection of the Sjogren’s Syndrome (SjS) has been recently developed (patent application No. 102015000020005 Italian Patent Office), based on PVX CVPs displaying a SjS diagnostic peptide. It has indeed been observed that serum antibodies recognize much less efficiently this soluble peptide if compared to the same peptide displayed on the viral scaffold so that the sensitivity of the assay is noticeably increased, preserving the specificity.

Potato virus X CVPs, displaying an antimicrobial decapeptide derived from an anti-idiotypic antibody that acts as a functional internal image of a microbicidal broad-spectrum yeast killer toxin, were active against *Staphylococcus aureus* and *Candida albicans*, as well as against the plant pathogens *Erwinia carotovora*, *Botrytis cinerea*, and *Fusarium oxysporum*. Moreover, they effectively protected plants from *Pseudomonas syringae* attacks (Donini et al., 2005).

Potato virus X CVPs carrying CPs fused to a single chain antibody specific to *Diuron* herbicide were purified from plant extracts by *Diuron*-based immune-capture, demonstrating their potential as tools for *in situ* applications including remediation of contaminated soils and waterways (Smolenska et al., 1998).

The fusion of GFP or mCherry protein to the PVX CP through the 2A peptide strategy allowed to follow infection progression throughout the plant, providing a non-invasive tool to the study of viral multiplication and spread (Santa Cruz et al., 1996, 1998; Roberts et al., 1997; Tilsner et al., 2013; Shukla et al., 2014c).

Potato virus X provides also opportunities for nanochemistry applications such as the construction of catalytic systems operating like metabolons in nature. A proof of concept has been published, in which the CVPs were constructed through the OVERCOAT^®^ system to display Lipase B, an enzyme derived from *Candida antarctica* known to be an efficient enantiospecific catalyst for chemical hydrolysis. The virus-anchored lipase molecules retained their catalytic activity toward the substrate p-nitrophenyl caproate in solution (Carette et al., 2007). Unlike manmade supported catalysts, these PVX-based biocatalysts were easily produced using plant machinery and, because the virus particle is made of many proteins, they intrinsically possessed multiple catalytic sites.

Recently, PVX nanoparticles have also been investigated as scaffold for the attachment of metal atoms in an attempt to develop new bioinorganic materials for nanoelectronics. Platinum atom clusters (Pt nanoparticles, Pt-NPs) were built selectively at one end of the virus helical structure by chemical reduction, yielding virions that resemble a push-pin with a platinum head and a virus needle (Drygin et al., 2013). These results highlight the specificity of the coordinate metal interaction with a complex polyvalent biological structure, thus providing insights on the mechanisms of Pt-NPs formation, important to understand the interaction of viral particles with ionic metals. Chimeric PVX particles have also been constructed displaying multiple copies of a peptide that is known to induce the formation of SiO_2_. By the conditions adopted for synthesis, isolated SiO_2_ spots of about 10 nm were formed along the surface of the chimeric virus. The not uniform SiO_2_ coating allowed additional immunogold labeling of the particles. The possibility to combine silicification with immunolabeling provides a method to develop triple-hybrid complex structures, with controllable composition and functional properties (Van Rijn et al., 2015).

## Conclusion

Overall, the knowledge of PVX, type-member of the *Potexvirus* genus, has evolved from basic molecular pathology to applications in biomedicine and material science.

The knowledge of plant-pathogen interactions and related silencing mechanisms has opened the way to the development of novel genetically-controlled strategies of plant infection, and through these to functional genomics and molecular farming.

By virtue of the innate versatility, PVX is now in the spotlight as a promising nanoobject for applications in vaccinology, drug targeting and delivery, diagnostic kits development and also as imaging reporter tool.

Last but not least, it is investigated as scaffold for enzymatic reactions or nanoelectronics.

For a long time, other plant viruses, mainly spherical or rigid rod-shaped, have been preferred to PVX for this kind of applications, but the data reviewed here clearly demonstrate that the flexuous mood of this nanoparticle is not a constraint rather a positive trait to be adequately exploited to respond to specific needs. Although many of the illustrated applications are still in their infancy, there is no reason to suppose they will not progress rapidly.

The shift of PVX from foe to friend has been made possible because this virus has been largely investigated in its innermost mechanisms. This cultural trail clearly witnesses how basic research can trigger a virtuous cycle of knowledge, removing existing interdisciplinary boundaries and opening up unimagined prospects, thus fuelling the development of innovative technologies.

### Conflict of Interest Statement

The authors declare that the research was conducted in the absence of any commercial or financial relationships that could be construed as a potential conflict of interest.
